# A Hybrid F–K Domain Feature Extraction and Enhancement Framework for Low-Frequency DAS Production-Logging Data: A Single-Well Field Case Study

**DOI:** 10.3390/s26134213

**Published:** 2026-07-03

**Authors:** Qiongqin Jiang, Yichen Zhong, Wenguang Song, Kai Zheng

**Affiliations:** School of Computer Science and Engineering, Guangdong Ocean University, Yangjiang 529500, China; jqq@gdou.edu.cn (Q.J.); 18823862840@stu.gdou.edu.cn (Y.Z.); 15013168099@stu.gdou.edu.cn (K.Z.)

**Keywords:** distributed optical fiber acoustic sensing, production logging, finite impulse response filtering, frequency–wavenumber domain, OCSVM, small-sample classification, flow velocity estimation

## Abstract

**Highlights:**

**What are the main findings?**

- A hybrid low-frequency DAS processing framework is proposed for distributed optical fiber production logging.
- PSO-optimized OCSVM, rule-based enhancement, and small-sample SVC refinement improve F–K domain V-shaped feature extraction.

**What are the implications of the main findings?**

- The enhanced F–K domain branches support interval-scale flow-velocity estimation from field DAS production-logging data.
- Six overlapping field validation windows show relative errors below 3.13%, while broader multi-well validation is still required.

**Abstract:**

Distributed optical fiber acoustic sensing (DAS) has become an important technology for production logging because it can record dense strain or strain-rate responses along an optical fiber under high-temperature, high-pressure, and corrosive downhole conditions. This single-well field case study investigated a hybrid low-frequency DAS processing framework for distributed optical fiber production logging. First, a finite impulse response (FIR)-based preprocessing step was used for low-pass smoothing before an F–K domain analysis. The DAS records were then transformed into the frequency–wavenumber (F–K) domain, where particle swarm optimization (PSO) was used to tune the nu parameter of a one-class support vector machine (OCSVM) for automatic feature extraction. Rule-based feature enhancement and a small-sample support vector classifier (SVC) were then applied to suppress residual F–K domain noise and retain the V-shaped features associated with upgoing and downgoing waves. Finally, linear regression was applied to the enhanced F–K domain branches to estimate the apparent propagation velocities and derive the flow velocity through the field interpretation relationship. The workflow was demonstrated using 15 s field DAS segments from an oil–water two-phase production well, and the six-window validation showed errors below 3.13% relative to the field-reference values. These results demonstrate the feasibility of the proposed workflow for the investigated well, but do not constitute general validation across different wells or acquisition conditions.

## 1. Introduction

Conventional logging instruments often exhibit significant measurement deviations in high-temperature, high-pressure, and corrosive environments. In contrast, distributed optical fiber logging offers resistance to a high temperature and pressure, corrosion tolerance, and dense spatial sampling, making it suitable for acquiring distributed optical fiber acoustic sensing (DAS) data under complex well conditions [1,2,3,4].

DAS interrogators launch coherent optical pulses into the fiber and analyze the Rayleigh backscatter generated by frozen-in microscopic density fluctuations in the glass. The axial strain changes the relative optical path lengths of scatterers within a finite gauge length, causing measurable phase changes in the backscattered field. Thus, DAS measures the dynamic axial strain or strain rate averaged over gauge-length sections, rather than the absolute acoustic intensity at unknown “distributed sensing points”. In production logging, pressure disturbances and flow-induced vibrations generate elastic waves that couple into the fiber; the measured response depends on coupling, the gauge length, the incidence direction, and the projection of the strain field along the fiber axis. Temperature changes mainly affect the refractive index and low-frequency drift, whereas dynamic acoustic and vibration sources mainly introduce strain [1,5,6,7,8].

Low-frequency DAS data in optical fiber logging are valuable because they preserve flow-related wavefield information over long sensing distances with a high channel density. They are not inherently immune to noise: optical phase noise, interrogator noise, poor cable coupling, common-mode disturbances propagating along the cable, pump and tool vibrations, temperature drift, and F–K domain artifacts may all contaminate the records. The purpose of the processing workflow developed here was to suppress unwanted frequency and wavenumber components while retaining coherent low-frequency features related to the wellbore flow [9,10].

DAS data in the time domain consist of continuous strain or strain-rate waveforms sampled at each channel or gauge-length location along the fiber. The temporal sampling rate determines the Nyquist frequency and the smallest time interval that can be resolved; in this study, the field records used for subsequent processing had a temporal sampling frequency of 5000 Hz after downsampling, which is sufficient for the later 0–150 Hz F–K domain analysis after anti-alias filtering [11,12].

In the spatial domain, signals are indexed by channel spacing and by gauge length. Channel spacing is the distance between adjacent reported samples, while the gauge length is the fiber interval over which phase changes are differenced or averaged. The number of recorded channels is determined by the sensing length and channel spacing, but the physical spatial resolution is also controlled by the gauge length and cable coupling. For the field case used here, the data were acquired with a Silixa DAS interrogator using an original temporal sampling frequency of 10,000 Hz and a spatial sampling rate of 2 samples/m, corresponding to a channel spacing of 0.5 m.

Despite these advantages, low-frequency DAS production-logging data remain difficult to interpret quantitatively. First, continuous monitoring produces large time–space matrices that require efficient preprocessing. Second, the useful flow-induced features may be weak and embedded in optical, mechanical, and coupling-related noise. Third, the manual interpretation of F–K domain features is time-consuming and operator-dependent. Traditional filters such as moving-average FIR filters, Butterworth filters, and wavelet denoising can suppress part of the noise, but they do not by themselves provide automated feature extraction or flow-velocity estimation. Intelligent classifiers such as OCSVM and SVC can support automatic recognition, but their performance depends on appropriate feature construction and limited labeled samples. Similar data-driven strategies have also been used for feature extraction, intelligent diagnoses, and measurement-signal analyses in complex engineering systems, supporting their potential value for noisy industrial monitoring data [13,14,15].

To address these issues, this study proposes a hybrid DAS signal-processing and interpretation framework that integrates FIR preprocessing, F–K-domain transformation, automated feature extraction, feature enhancement, and velocity estimation. The main contributions are as follows:

1. A low-frequency DAS preprocessing workflow was defined, including FIR-based smoothing, anti-alias filtering, and downsampling.
2. A PSO-optimized OCSVM strategy was developed for automatic feature extraction in the F–K domain.
3. A rule-assisted and small-sample SVC feature enhancement strategy was introduced to suppress F–K domain streak noise and sparse residual noise.
4. A field-oriented flow-velocity estimation workflow was constructed by linking the DAS response, F–K domain branch slopes, apparent propagation velocity, and production-logging velocity interpretation.

The remainder of this paper is organized as follows. Section 2 describes the materials and methods, including the DAS acquisition setting, low-frequency preprocessing, F–K domain transformation, PSO-optimized OCSVM feature extraction, and feature enhancement. Section 3 reports the field validation and velocity-estimation results. Section 4 discusses limitations and future work, and Section 5 concludes the paper.

## 2. Materials and Methods

### 2.1. DAS Data Acquisition and Field Setting

**Figure 1:**
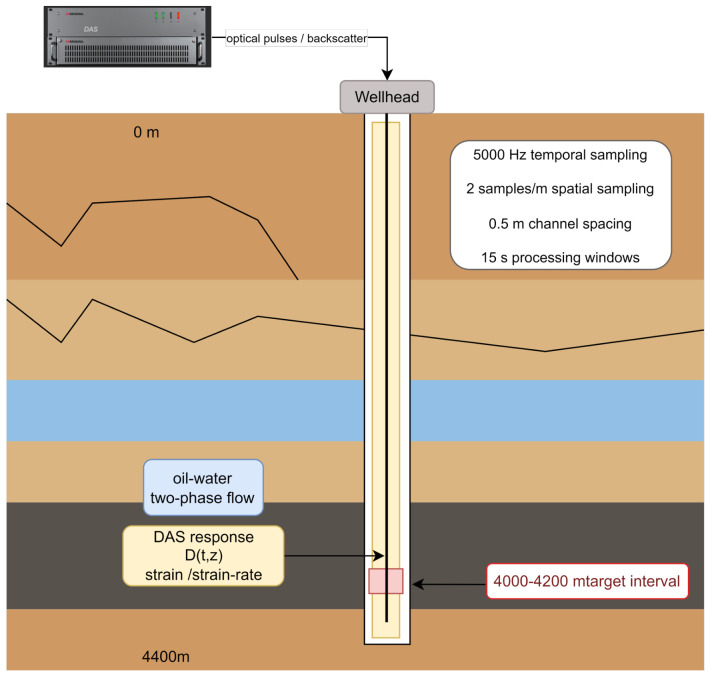
Schematic of the DAS production-logging acquisition system.

Figure 1 illustrates the DAS production-logging configuration used in this study. A surface interrogator sends coherent optical pulses into a sensing fiber deployed along the wellbore. Flow-induced pressure and vibration disturbances couple into the fiber and produce measurable axial strain responses. In this study, the DAS data were acquired using a Silixa DAS interrogator at an original temporal sampling frequency of 10,000 Hz. The spatial sampling rate was 2 samples/m, corresponding to a channel spacing of 0.5 m. The DAS gauge length used during acquisition was 10 m. The fiber sensing length was approximately 4400 m, and the target interval used for interpretation was 4000–4200 m. The main acquisition and processing configuration parameters are summarized in Table 1.

### 2.2. Low-Frequency DAS Preprocessing

The DAS data used for optical fiber logging are stored as two-dimensional arrays D(t,z), where *t* denotes time and *z* denotes the channel position or gauge-length location along the fiber. Each matrix element stores the measured axial strain or strain-rate response after interrogator demodulation, not a generic amplitude. The rows correspond to temporal samples, the columns correspond to spatial channels, and the F–K transform later used in this paper was applied to this time–space matrix [16,17].

**Figure 2:**
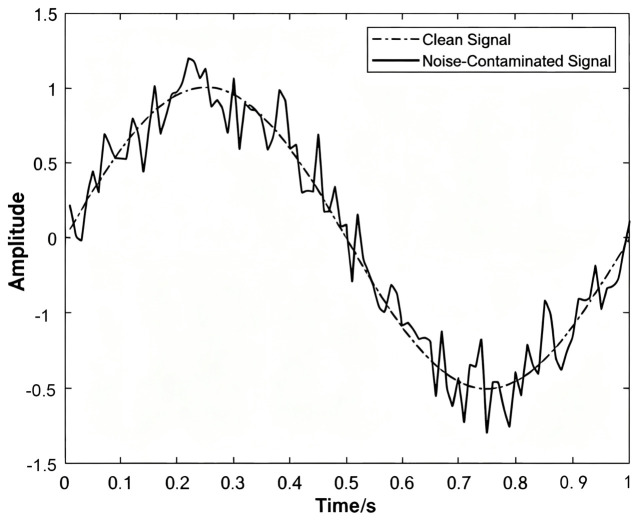
Time–response plot of simulated clean and noisy DAS signals. The vertical axis is the normalized strain-rate response, and the example is used only to illustrate filtering behavior.

In Figure 2, the horizontal axis represents the time in seconds, and the vertical axis denotes the normalized DAS response. Two simulated signal segments are shown: a clean sinusoidal signal and the same signal contaminated with additive white noise. The rapid fluctuations in the noisy signal illustrate high-frequency components that should be attenuated before the data are downsampled. Filtering and downsampling are separate operations: anti-alias filtering suppresses components above the target Nyquist frequency, and only after filtering can samples be safely decimated. For a low-frequency DAS production-logging analysis, FIR filters are attractive because they have a stable finite memory, the linear-phase designs are straightforward, and their convolutional implementation is computationally simple [4,10,18,19].

**Figure 3:**
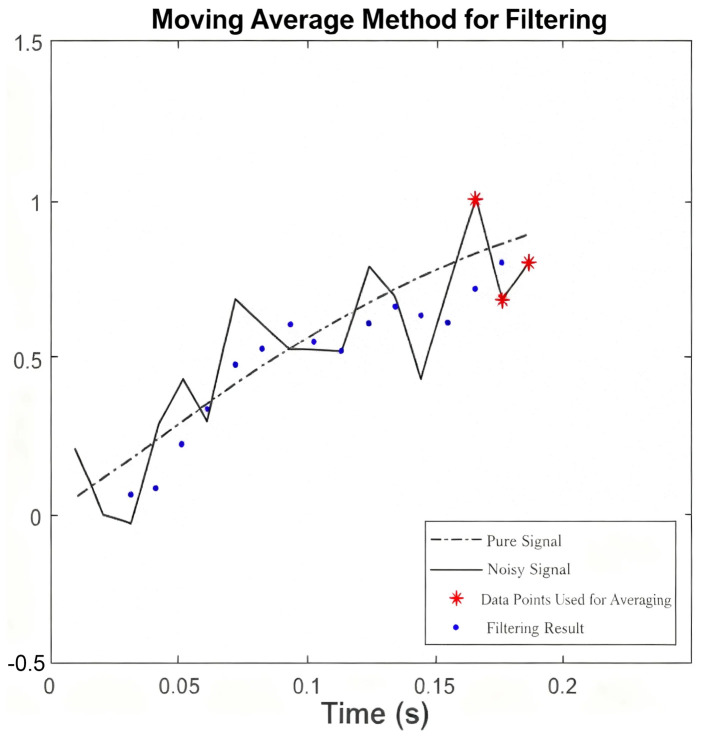
Processing performance of the three-point FIR moving-average filter on a simulated DAS signal. The vertical axis is the normalized strain-rate response.

The noise-contaminated signal exhibited oscillations above and below the clean signal. In this illustrative example, random high-frequency fluctuations were mitigated by computing the average of every three adjacent samples, thereby yielding the filtered output. The three-point moving average is a simple FIR filter, as illustrated in Figure 3, and is used here as the preprocessing step before downsampling and F–K domain feature extraction.

The three-point moving-average filter was selected as a lightweight local-smoothing operation rather than as an optimal frequency-selective denoising filter. Its short impulse response requires only three multiplications and additions per output sample and introduces limited temporal averaging of the low-frequency signal. The objective of this step was to reduce short-period fluctuations before F–K domain processing while avoiding an unnecessarily long smoothing window that may alter transient wavefield features.

The moving-average filter is a special case of an FIR filter. In this study, FIR preprocessing was implemented as a three-point moving-average filter applied along each temporal DAS trace. For a discrete DAS trace x[n] at sample index *n*, the filtered output y[n] is computed as the arithmetic mean of the three adjacent samples x[n−1], x[n], and x[n+1], with each sample weighted by 1/3, and is defined as Equation (1):

(1) $$y\left[n\right]=\frac{1}{3}\left(x[n-1]+x[n]+x[n+1]\right).$$

This operation corresponds to the finite impulse response kernel *h* shown in Equation (2):

(2) $$h=\left[\frac{1}{3},\frac{1}{3},\frac{1}{3}\right].$$

For an *L*-point moving-average filter, where *L* denotes the filter length, the frequency response $H_{L}\left(e^{j\omega }\right)$ at angular frequency ω is expressed with *j* as the imaginary unit:

(3) $$H_{L}\left(e^{j\omega }\right)=e^{-j\omega (L-1)/2}\frac{\sin(L\omega /2)}{L\sin(\omega /2)}.$$

Increasing *L* narrows the effective passband and produces stronger smoothing, but it also averages the signal over a longer temporal interval and increases the number of arithmetic operations per output sample. Compared with five- and seven-point alternatives, the three-point kernel is the least aggressive and the computationally least expensive smoothing choice. It was selected because this preprocessing stage is intended only to reduce the sample-to-sample fluctuations before the F–K domain analysis, rather than to provide sharp frequency-band separation. The three-point kernel is not claimed to be universally optimal, and longer filters may be preferable under different noise conditions. Filter lengths of 3, 5, and 7 samples were evaluated while all other processing parameters were kept unchanged.

The purpose of this step was to suppress short-period random fluctuations before F–K domain feature extraction. It was used as a conventional smoothing operation rather than as a deep-learning component.

**Figure 4:**
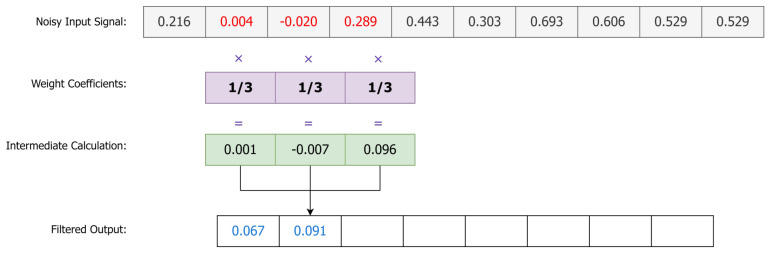
FIR filtering procedure implemented as a one-dimensional convolution.

The convolution operation in the FIR preprocessing method applies a single DAS trace to the FIR impulse response to generate a filtered DAS trace; this is a single-trace filtering operation. In DAS signal processing, convolution is commonly used to represent the response of a linear time-invariant filter [20,21]. The three-point FIR kernel multiplies adjacent samples from the same trace by coefficients (1/3,1/3,1/3) and sums the result. The physical meaning is local temporal smoothing before F–K domain feature extraction, which suppresses short-period fluctuations while preserving the low-frequency components used in production-logging interpretation. The filtering process is illustrated in Figure 4.

In the FIR filtering method, the input signal x(t) is a single DAS channel trace, and h(t) is the FIR filter impulse response. Their convolution is expressed as y(t)=x(t)∗h(t), with its mathematical definition given in Equation (4).

(4) $$y\left(t\right)=\left(x*h\right)\left(t\right)=\int_{-\infty }^{\infty }x\left(\tau \right)h\left(t-\tau \right)\;d\tau .$$

In Equation (4), τ is the integration variable and *t* is the time variable of the DAS output signal. Physically, the DAS input signal x(τ) acts at time τ, and the system response h(t−τ) quantifies its influence at time *t*.

**Figure 5:**
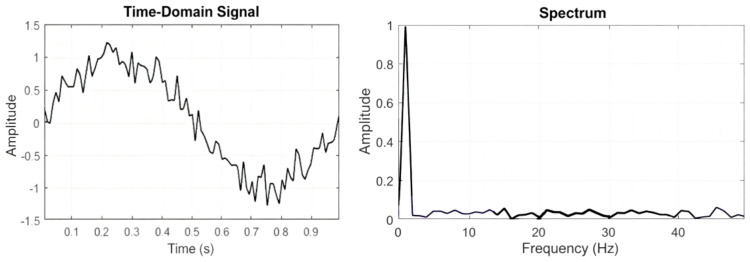
Time- and frequency-domain views used to select the low-frequency passband. The **left panel** shows the time-domain signal, and the **right panel** shows the spectral energy before final downsampling.

A crucial property of convolution in FIR filtering is that convolution in the time domain is equivalent to multiplication in the frequency domain. This property is closely related to filtering, as the objective of filtering is often to extract or eliminate specific frequency components from DAS signals [22,23,24]. To filter noise from a noisy DAS signal, the frequency spectrum of the signal should first be examined to identify the frequency range of the noise. A suitable FIR filter can then be selected to attenuate noise components within these frequency ranges. Figure 5 visualizes the signal in both the time and frequency domains.

As shown in Figure 5, the dominant low-frequency energy in the illustrative signal was concentrated below approximately 3 Hz, while higher-frequency components were treated as noise for this example. This cutoff is data-dependent and should be selected from the spectrum of each logging interval. The frequency-domain view was used only to diagnose the passband and verify attenuation; the three-point FIR operation itself was performed in the time domain.

When a filter is designed and applied in the frequency domain, the filtered spectrum is transformed back to the time domain by an inverse Fourier transform. In the proposed workflow, however, the preprocessing filter is implemented directly as time-domain convolution, so a fast Fourier transform (FFT) is not required for this anti-alias filtering step. The later two-dimensional fast Fourier transform (2D FFT) is a distinct operation used for F–K domain feature extraction and velocity estimation. Thus, filtering, downsampling, and F–K domain feature extraction are three separate steps [23].

A higher-order linear-phase FIR filter could provide a sharper transition band and stronger stopband attenuation. Other alternatives, such as Butterworth filtering, Savitzky–Golay smoothing, and wavelet denoising, may also be appropriate for different noise conditions. However, the present study did not perform a controlled comparison among filter designs.

**Figure 6:**
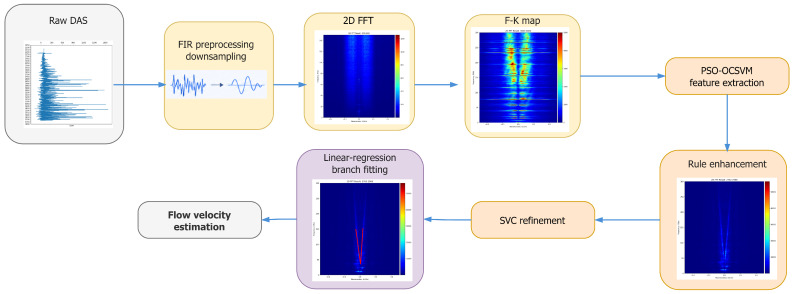
Overall signal-processing workflow for the proposed low-frequency DAS production-logging framework.

Figure 6 summarizes the proposed DAS processing and velocity-estimation workflow. The workflow is organized as a sequential interpretation chain: FIR preprocessing and downsampling reduce high-frequency fluctuations and data volume; the selected time–space window is transformed to the F–K domain; PSO–OCSVM extracts candidate V-shaped wavefield features; rule-based enhancement removes axis-parallel streak artifacts; SVC refinement suppresses sparse residual noise; and linear regression of the enhanced F–K domain branches provides the apparent propagation velocities used for the flow-velocity calculation.

During DAS processing, the two-dimensional dataset is partitioned along the wellbore depth. Each channel trace is first filtered, and downsampling is then achieved by keeping one sample every *M* samples [24,25]. If the original sampling rate is $f_{s}$, the downsampled sampling rate is $f_{s}^{\prime }$, the original number of samples is *N*, and the downsampled number of samples is $N^{\prime }$, then the relationship is expressed by Equation (5):

(5) $$f_{s}^{\prime }=\frac{f_{s}}{M},\;N^{\prime }=\frac{N}{M}.$$

This implies that, if the original signal has a sampling rate of $f_{s}$, the downsampled data volume is reduced to 1/M of the original data volume. In the field case, the original DAS records were acquired at 10,000 Hz. Temporal downsampling was performed using a factor of M=2, resulting in the 5000 Hz sampling frequency used for subsequent F–K domain processing. Anti-alias filtering was applied as part of the temporal decimation step.

### 2.3. F–K Domain Transformation

DAS data spanning multiple 15 s intervals were continuously acquired from an oil–water two-phase production well approximately two hours after well opening. The original temporal sampling frequency was 10,000 Hz, and the processing sampling frequency after temporal downsampling was 5000 Hz. The spatial sampling rate was 2 samples/m, corresponding to a channel spacing of 0.5 m, and the fiber sensing length was approximately 4400 m. The target perforation interval was located at 4000–4200 m. The DAS response matrix from this interval was selected and transformed from the time–space domain to the F–K domain using 2D FFT [26,27,28].

Each 15 s segment contains approximately 75,000 temporal samples per channel. With a spatial sampling rate of 2 samples/m, the 4000–4200 m target interval contains approximately 400 spatial samples. Thus, each selected time–space matrix used for the F–K domain analysis had an approximate size of 75,000×400 before any preprocessing or cropping.

The original DAS data capture response information along the entire sensing fiber. Because perforations and producing zones are spatially localized, the workflow first selects the interpretation interval and then transforms the corresponding time–space matrix from the *t*–*z* domain to the frequency–wavenumber (F–K) domain for a subsequent wavefield analysis. This 2D FFT is not part of the FIR smoothing operation discussed in the preprocessing subsection; it is a later step used to estimate wave slopes and velocities.

**Figure 7:**
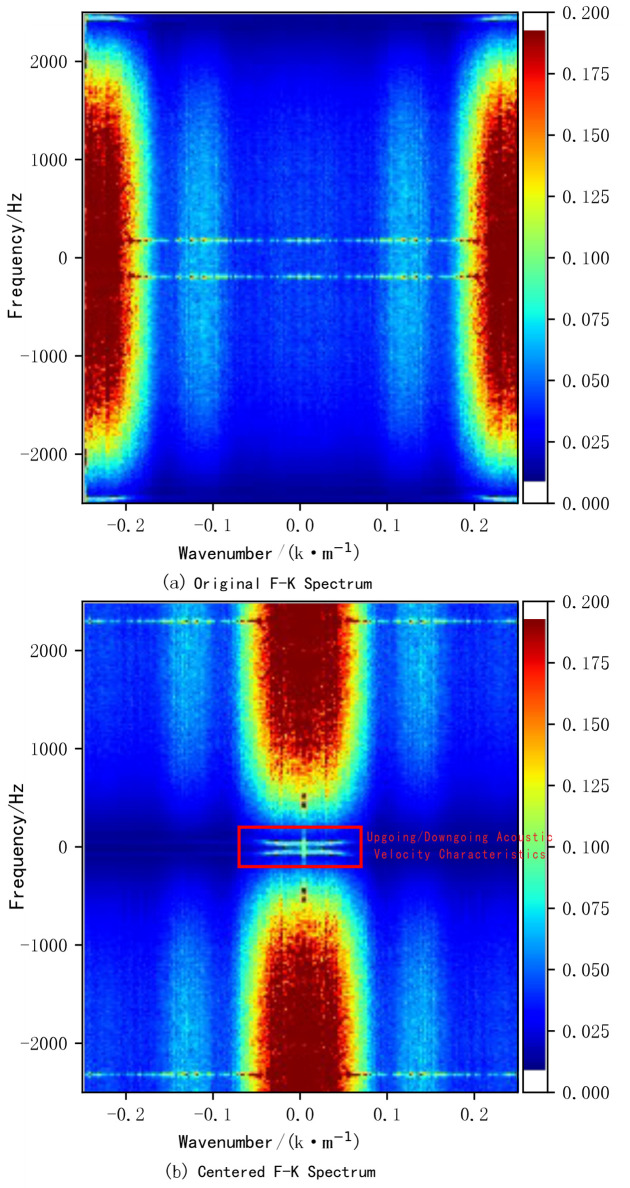
F–K domain representation of the selected DAS time–space matrix: (**a**) unshifted spectrum after 2D FFT, displayed with signed coordinate labels; (**b**) centered F–K domain spectrum after FFT shift, where the zero-frequency and zero-wavenumber coefficients are moved to the center of the spectral array.

The selected time–space DAS matrix from the 4000–4200 m interval was first transformed using a 2D FFT, producing the unshifted spectrum *A*, as shown in Figure 7a. Although zero is displayed at the center of the signed frequency and wavenumber axes in Figure 7a, this tick denotes only the coordinate origin; the FFT coefficients in *A* remain in their native unshifted array ordering. An FFT-shift operation was subsequently applied along both dimensions to obtain $A^{\prime }=\mathrm{fftshift}\left(A\right)$, in which the zero-frequency and zero-wavenumber coefficients are located at the center of the spectral array, as shown in Figure 7b. In Figure 7b, the horizontal axis represents the centered wavenumber, and the vertical axis represents the centered frequency.

**Figure 8:**
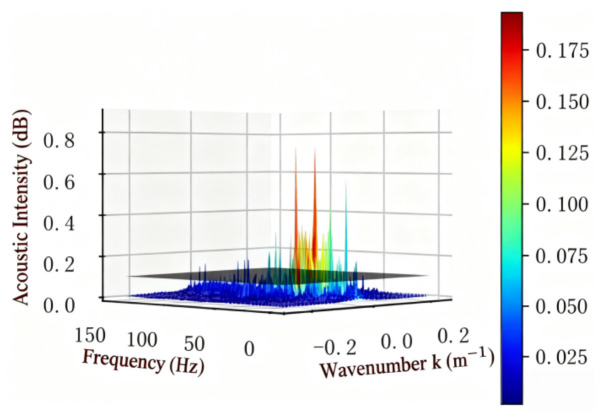
Three-dimensional visualization of the truncated F–K domain response. The black square denotes the truncation plane used to display the selected response range. The vertical axis represents the relative acoustic intensity in dB.

After FFT shifting, the useful upgoing and downgoing wavefield components appear as approximately symmetric linear branches in the centered F–K domain spectrum. Because the production-logging response of interest is concentrated in the low-frequency band, the 0–150 Hz portion of $A^{\prime }$ was extracted for a subsequent feature analysis. The extracted 0–150 Hz portion of $A^{\prime }$ is hereafter denoted by *F*. The 0–150 Hz band was selected empirically based on field experience with the investigated well and inspection of its F–K domain response. For this oil–water two-phase production well, most of the identifiable DAS response associated with the wellbore flow was observed within this frequency range, where the upgoing and downgoing wavefield components formed a visible V-shaped feature. Restricting the analysis to 0–150 Hz retained the principal flow-related wavefield features while reducing unrelated higher-frequency components. This frequency range is specific to the present field case and may require adjustment for other wells, production conditions, or acquisition configurations. For visualization, the truncated F–K domain response is displayed as a three-dimensional surface in Figure 8, where the vertical axis represents the relative acoustic intensity in dB. The truncated F–K-domain data contain two approximately oblique response branches associated with the upgoing and downgoing wavefield components. Their V-shaped distribution is more clearly visible in the later two-dimensional staged feature map. This observation motivated the following threshold-count representation and OCSVM-based feature extraction [25,29]. In this paper, “density” refers to the number of F–K samples whose response magnitudes fall within a given threshold interval, rather than physical density.

In the three-dimensional representation, the black plane represents a schematic decision boundary used to separate lower-count response intervals that include the V-shaped wave features from higher-count background intervals. The resulting boundary was used to extract the target features by thresholding before rule-based enhancement and SVC refinement [30].

### 2.4. PSO-Optimized OCSVM Feature Extraction

Forty datasets acquired at different time intervals during the well-opening period were used to train the OCSVM model. Each dataset was further divided into 200 m spatial intervals before F–K transformation. The 0–150 Hz portion of each F–K representation was then extracted to construct the threshold-count statistical features used for OCSVM training, as defined in Equations (6) and (7).

(6) $$T_{z}=\min\left(|F|\right)+\frac{z-1}{N_{T}-1}\left[\max(|F|)-\min(|F|)\right].$$

(7) $$\mathrm{count}\left(k\right)=\sum_{i=1}^{m}\sum_{j=1}^{n}1\left(T_{k}\leq \left|F_{ij}\right|<T_{k+1}\right).$$

In Equation (6), $T_{z}$ denotes the *z*-th threshold value, $N_{T}$ is the number of threshold points, and |F| indicates the modulus of the F–K domain data. In Equation (7), count(k) is the number of data points in the *k*-th interval, *m* and *n* are the row and column dimensions of Matrix *F*, and $F_{ij}$ denotes an element of Matrix *F*.

The statistical list computed using Equations (6) and (7) was employed to train an OCSVM model with a linear kernel function. Particle swarm optimization (PSO), a population-based search algorithm in which candidate solutions update their positions according to individual and global best solutions, was used to optimize the OCSVM hyperparameter ν [31]. In the present implementation, PSO is used to optimize only the OCSVM parameter (ν), while the kernel is fixed as linear. Accordingly, the optimization is limited to a one-dimensional parameter search, although the population-based procedure still introduces an additional computational cost. The PSO fitness criterion was the classification accuracy of the OCSVM on a test dataset acquired from an offshore field block. For each candidate value of ν, the OCSVM was trained using the threshold-count features defined in Equations (6) and (7) and then evaluated on the test dataset. The classification accuracy was calculated as

(8) $$\mathrm{Accuracy}\left(\nu \right)=\frac{N_{\mathrm{correct}}\left(\nu \right)}{N_{\mathrm{test}}},$$

where $N_{\mathrm{correct}}\left(\nu \right)$ is the number of correctly classified test samples and $N_{\mathrm{test}}$ is the total number of test samples. The “best separation” criterion refers to the candidate value of ν that produced the highest test-set classification accuracy. The selected value was ν=0.954681, corresponding to an accuracy of 96.88%. The linear kernel was selected to provide a simple decision boundary without introducing additional kernel parameters under the limited-data setting. The specific parameter configurations and optimization results are summarized in Table 2.

In OCSVM, ν∈(0,1] is a model hyperparameter that controls the upper bound on the fraction of training errors and the lower bound on the fraction of support vectors. The symbol ν denotes the OCSVM hyperparameter, whereas *k* and $v_{\mathrm{app}}$ denote the wavenumber and the apparent propagation velocity, respectively. Only ν was optimized because the OCSVM employed a linear kernel. The RBF kernel-width parameter γ is not applicable to the selected linear-kernel model.

Because only one scalar parameter is optimized, a deterministic one-dimensional grid search could provide a simpler and more reproducible alternative. Direct statistical threshold selection could also reduce the computational cost. The relative accuracy and computational efficiency of these alternatives remain to be evaluated under consistent field conditions.

Using the linear kernel and optimized value of ν, the OCSVM assigns one of two class labels to each sample: outputs of “−1” indicate that the data point belongs to the normal class, while outputs of “+1” denote that the data point falls into the anomalous class.

**Figure 9:**
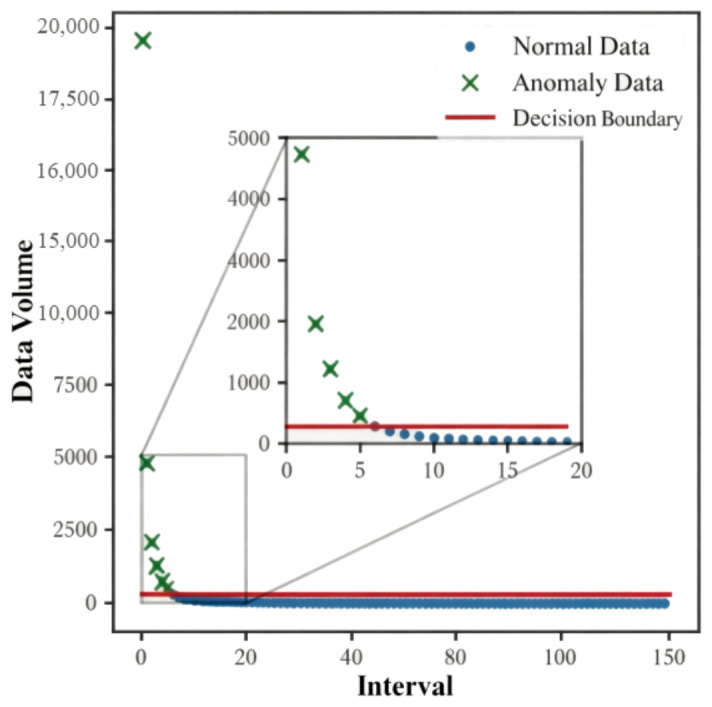
OCSVM classification results.

The classification results of the OCSVM are shown in Figure 9. In this figure, “interval” denotes the threshold interval of the normalized F–K response magnitude defined by Equation (6). The results demonstrate that the decision boundary separates high-count background intervals from lower-count feature-specific intervals, allowing the V-shaped wavefield features to be isolated.

In Figure 9, “normal” and “anomaly” denote OCSVM classes of threshold-count intervals and do not refer to well operating conditions.

**Figure 10:**
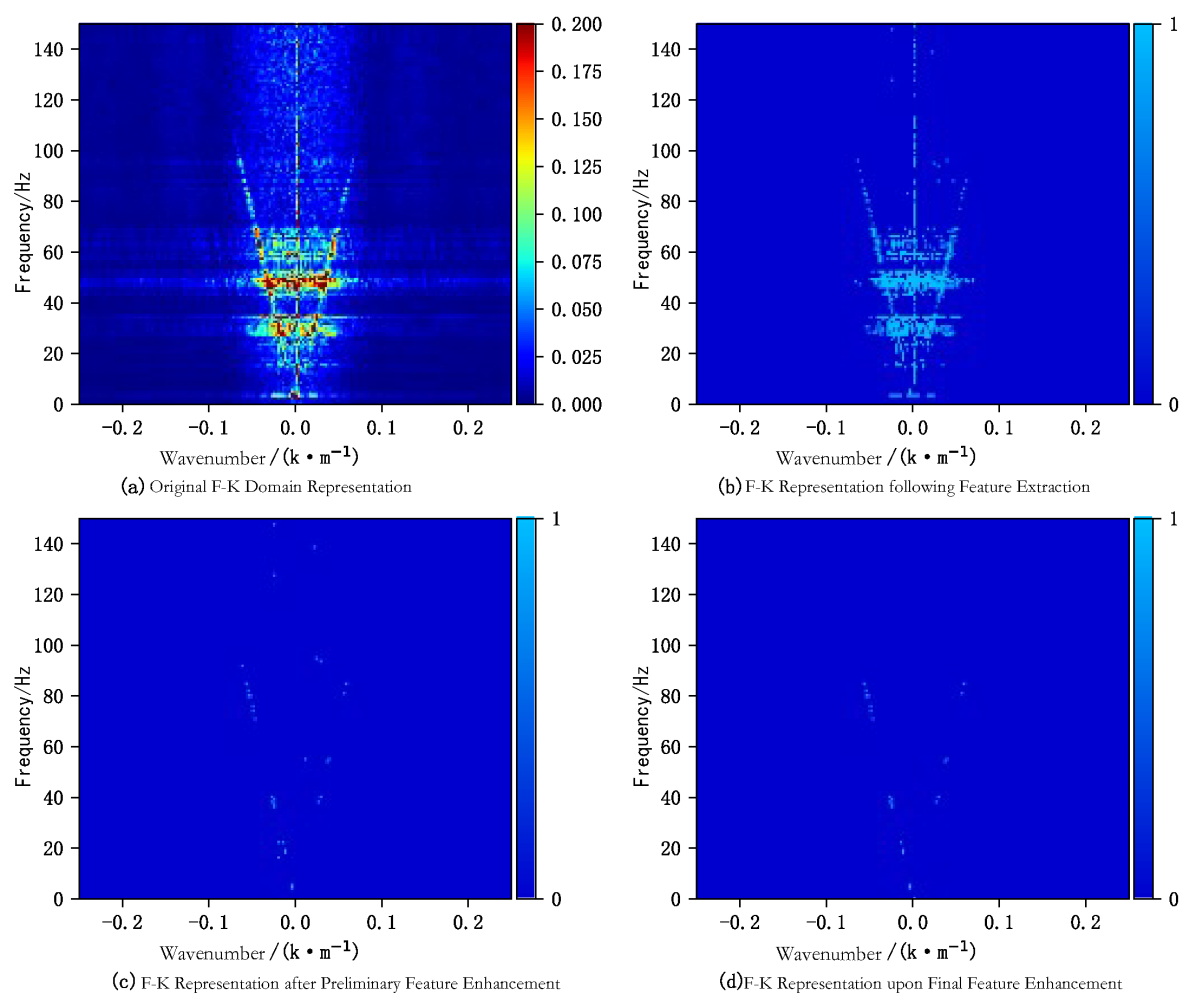
Staged F–K domain feature extraction and enhancement results: (**a**) original F–K domain representation; (**b**) F–K domain representation following feature extraction; (**c**) F–K domain representation after preliminary feature enhancement; and (**d**) F–K domain representation upon final feature enhancement.

The decision boundary computed by the OCSVM is applied as a filtering threshold to perform binarization processing on Matrix $A^{\prime }$, resulting in a binary matrix *B*. The visualization result of this matrix is shown in Figure 10b.

Figure 10 shows the staged effect of the proposed F–K domain enhancement workflow. The low-frequency F–K domain feature map in Figure 10a contains both V-shaped wavefield features and axis-parallel artifacts. After OCSVM thresholding, Figure 10b retains candidate V-shaped pixels, but also includes residual streaks and isolated points. Rule-based enhancement removes most axis-parallel artifacts, as shown in Figure 10c. Finally, SVC refinement further suppresses sparse residual noise near the branches, producing the cleaner feature map shown in Figure 10d, which is used for branch fitting and velocity estimation.

Based on the DAS acquisition and processing configuration summarized in Table 1, the processing evidence chain was organized as follows: raw F–K domain map, shifted F–K domain map, 0–150 Hz truncated F–K domain feature map, OCSVM extraction, rule enhancement, and SVC refinement. The corresponding figures and their roles are summarized in Table 3.

### 2.5. Feature Enhancement

**Figure 11:**
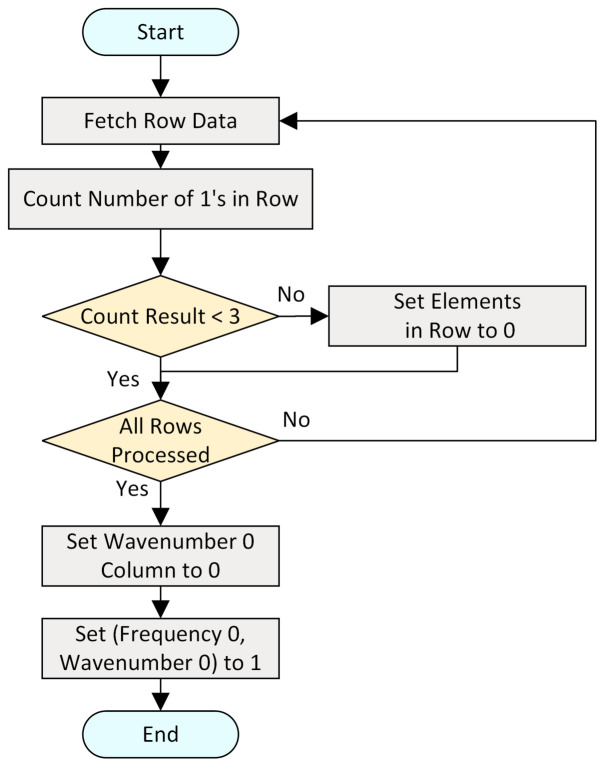
Feature enhancement workflow.

As observed in Figure 10b, interference signals persist after feature extraction. In the F–K domain, these interferences are classified into three practical types: horizontal streaks approximately parallel to the frequency axis, vertical streaks around the zero wavenumber, and sparse isolated points around the V-shaped feature. Horizontal streaks mainly affect row-wise statistics, vertical streaks can cause near-zero-slope artifacts in velocity fitting, and sparse points increase the regression error [32,33]. The first two classes are removed by explicit statistical rules, and the remaining sparse noise is refined using the SVC model. The feature enhancement rules are illustrated in Figure 11.

Starting from the binary matrix *B* generated by OCSVM thresholding, three empirical rules were applied:

1. For each row of Matrix *B*, the number of active pixels was counted. If the number was not less than 3, the entire row was set to 0; otherwise, the row was retained. This operation removes horizontal interference approximately parallel to the frequency axis.
2. The column corresponding to the zero wavenumber was set to 0 to avoid zero-slope artifacts during velocity fitting.
3. The point corresponding to the zero frequency and zero wavenumber was reset to 1 as an anchor point for subsequent separation of the two V-shaped branches, resulting in Matrix $B^{\prime }$.

These rules are based on the observed morphological differences between the target V-shaped wavefield branches and the dominant artifacts in the present F–K domain maps. The wavefield components retained for velocity estimation appear as oblique linear branches, whereas the dominant structured artifacts appear as horizontal streaks or responses concentrated near the zero wavenumber. Accordingly, the rule-based operation serves as a coarse morphology-guided stage for removing the dominant axis-parallel artifacts before SVC refinement. Its applicability is therefore limited to F–K maps with a similar artifact morphology. In particular, the not-less-than-3 threshold was selected from the observed row-wise distribution of the present binarized F–K domain maps and is consistent with the decision logic shown in Figure 11. The rule thresholds are empirical values selected for the present dataset and may require recalibration under different acquisition parameters, F–K domain resolutions, or noise conditions. The SVC model is then applied to Matrix $B^{\prime }$ to identify and remove sparse residual noise points near the V-shaped features, further refining the feature boundaries after the coarse rule-based denoising.

The preliminary feature enhancement results are shown in Figure 10c. A comparison between Figure 10b,c demonstrated that, after preliminary feature enhancement, the V-shaped feature reflecting upgoing and downgoing waves became more distinct, while most interference parallel to the F–K axes was eliminated.

As illustrated in Figure 10c, a limited number of irregular noise data points persisted around the V-shaped feature, necessitating further processing to mitigate their impact on the velocity calculations. A supervised learning support vector classifier (SVC) model was employed to learn the mapping relationship between the input features and the output labels, thereby enabling accurate predictions on unseen data and achieving enhanced feature refinement [27].

**Figure 12:**
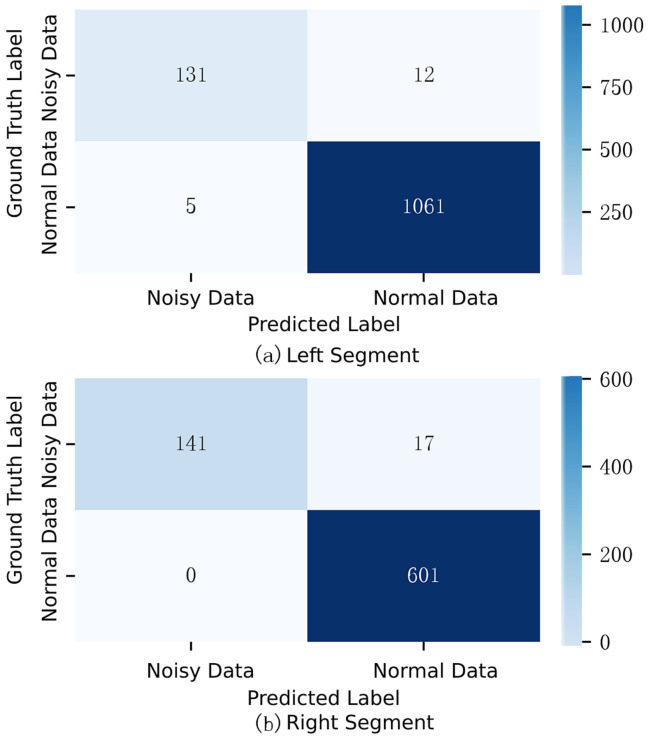
Confusion matrices for SVC classification of the left and right V-shaped branches: (**a**) left branch; (**b**) right branch.

The SVC training samples were constructed from the preliminarily enhanced V-shaped F–K domain feature maps. A total of 32 V-shaped feature datasets were used. Each V-shaped feature was divided into left and right branches at the zero-wavenumber position, and the two branches were trained separately. The trained SVC was then applied to Matrix $B^{\prime }$ to identify sparse residual noise points around the V-shaped branches and remove them before linear regression. The confusion matrices for SVC classification are shown in Figure 12. The derived classification metrics are listed in Table 4. The processed results are presented in Figure 10d.

## 3. Results

### 3.1. Field Case Validation

The proposed workflow was applied to field DAS data from the investigated oil–water two-phase production well. The field reference velocities used for comparison were obtained from production-operation records; the DAS-based calculation provides distributed interval-scale estimates that complement sparse conventional point measurements and operational records.

### 3.2. Field Preprocessing and Feature Extraction Results

DAS systems typically perform continuous long-term monitoring of the downhole conditions, resulting in substantial data volumes. In this field case, the acquired data were processed as 15 s windows. The 2D FFT algorithm decomposes the two-dimensional discrete Fourier transform (DFT) into two sequential one-dimensional DFT operations. In this study, F–K transformation was conducted by applying the 2D FFT to each selected 15 s time–space window and shifting the zero-frequency and zero-wavenumber components to the center of the two-dimensional spectrum.

The volume of characteristic data reflecting the upgoing and downgoing apparent propagation velocities is comparatively small relative to the overall F–K domain data. By selecting an appropriate threshold on the response magnitude, the characteristic data can be isolated, thereby accomplishing feature extraction. This process simultaneously reduces the data volume and computational complexity, facilitating further analyses and interpretation [10,34,35]. A statistical analysis is performed across response-magnitude intervals, and a decision boundary is computed using the OCSVM to distinguish background intervals from feature-bearing intervals.

### 3.3. Field Feature Enhancement and Velocity Estimation Results

Following feature extraction from the F–K domain data, a limited number of noise points remain irregularly distributed around the extracted features, adversely affecting the accuracy of wave-slope calculations and later velocity estimation. Denoising is applied to the initially extracted features. By analyzing the distribution patterns of features and noise within the F–K domain data, rule-based processing removes axis-parallel streaks, while the SVC removes sparse residual points [13,36,37].

The staged enhancement shown in Figure 10 directly improves the stability of branch selection for linear regression. The OCSVM stage provides the initial candidate pixels, the rule-based stage removes structured artifacts, and the SVC stage suppresses sparse outliers near the branches. The final branches in Figure 10d were used for branch fitting, uncertainty analysis, and velocity estimation.

Through the sequential processing stages of preprocessing, feature extraction, and feature enhancement, the flow velocity was calculated. In the F–K domain, a coherent linear event satisfies

(9) $$f=v_{\mathrm{app}}k+b,$$

where *f* is the frequency, *k* is the wavenumber, *b* is the fitted intercept, and the regression slope $v_{\mathrm{app}}$ represents the apparent propagation velocity of the branch. Linear regression was applied to the enhanced left and right branches to estimate the upgoing and downgoing apparent propagation velocities, $v_{\mathrm{up}}$ and $v_{\mathrm{down}}$.

The uncertainty of each fitted F–K branch slope was quantified using the standard error and 95% confidence interval of the ordinary least-squares estimate. For the regression model $f_{i}=v_{\mathrm{app}}k_{i}+b+\varepsilon_{i}$, the slope standard error was calculated as

(10) $$SE\left({\hat{v}}_{\mathrm{app}}\right)=\sqrt{\frac{\sum_{i=1}^{n}{\left(f_{i}-{\hat{f}}_{i}\right)}^{2}/\left(n-2\right)}{\sum_{i=1}^{n}{\left(k_{i}-\bar{k}\right)}^{2}}}.$$

The corresponding 95% confidence interval was calculated as

(11) $${\hat{v}}_{\mathrm{app}}\pm t_{0.975,n-2}SE\left({\hat{v}}_{\mathrm{app}}\right).$$

For the representative field window, the negative-wavenumber branch yielded a slope of −1559.41 m/s, with a 95% confidence interval of [−1582.38,−1526.44] m/s. The positive-wavenumber branch yielded a slope of 1591.33 m/s, with a 95% confidence interval of [1588.76,1593.90] m/s. These statistics quantify uncertainty in the apparent propagation velocities obtained from the F–K branch fitting. They do not represent the complete uncertainty of the final flow-velocity estimate, which also depends on the field-calibration relationship. The branch-fitting uncertainty results are summarized in Table 5.

The reported flow velocity is obtained from the calibrated field interpretation relationship

(12) $$v_{f}=G\left(v_{\mathrm{up}},v_{\mathrm{down}},\theta \right),$$

where $v_{f}$ is the interpreted flow velocity and θ denotes field calibration factors associated with the well completion, coupling condition, and operational reference records. Because the calibration factors are confidential, validation was limited to comparisons between the DAS-derived estimates and field-reference velocities in six overlapping depth windows. In this case study, the calculated flow velocity was 15.96 m/s, which approximates the 16 m/s recorded by field development personnel. A comparison between the model outputs across different wellbore intervals and field records is presented in Table 6.

The sensitivity of the estimated velocity to the FIR filter length is summarized in Table 7.

The relative error increased with the filter length. The three-point filter achieved the lowest error and was therefore retained in the proposed method.

In the six overlapping validation windows, the relative error ranged from 0.08% to 3.13%. This result indicates that DAS can supplement conventional production records by providing distributed interval-scale velocity estimates along the wellbore, although the present validation should not be interpreted as proof of the general accuracy across wells, reservoirs, or acquisition configurations.

## 4. Discussion

The proposed workflow combines magnitude-based statistical extraction, morphology-guided artifact removal, and supervised refinement. Fixed thresholding would reduce the computational cost, but may be sensitive to interval-dependent amplitude variations. Hough-transform and morphological methods provide alternative means of extracting linear branches, whereas nonlinear OCSVM kernels may represent more complex feature boundaries. These alternatives define a comparison space for assessing the robustness, computational cost, and transferability.

This study has several limitations. The primary limitation lies in the patterned rules used in the feature enhancement stage. These rules are based on the observed morphological differences between coherent V-shaped wavefield branches and axis-parallel artifacts in the F–K domain data used in this study. Specifically, the rule-based operation assumes that the wavefield components retained for velocity estimation appear as oblique linear branches, whereas the dominant interference appears as horizontal streaks or responses concentrated near the zero wavenumber. The rule-based operation serves as a coarse morphology-guided stage for removing the dominant axis-parallel artifacts before SVC refinement. Its applicability is therefore limited to F–K maps with a similar artifact morphology. Although this approach achieved good results in this field case, its generalizability may be limited. If new noise patterns emerge that do not conform to these preset rules, such as oblique coherent streaks, common-mode cable noise, cycle-skipping artifacts, or sparse scatter-like points caused by poor coupling, the rule-based method may fail. In addition, the rule thresholds are empirical and may require recalibration under different acquisition parameters, F–K domain resolutions, or noise conditions. Although the following support vector classifier (SVC) model can remediate this to some extent, its effectiveness depends on the quantity and characteristics of the residual noise remaining after initial rule-based denoising. Consequently, the method remains dependent on prior knowledge of the dominant artifact morphology.

A second limitation is the restricted validation scope. The available field data came from a single confidential oil–water two-phase production well, and the six reported intervals are spatially overlapping windows rather than independent wells or independent reservoir settings. The reference velocities are operational field records, and detailed completion, production, pressure, temperature, and calibration metadata cannot be disclosed. Thus, the present results should be interpreted as validation of the workflow in this single-well field case study, not as a general performance guarantee.

Another limitation involves the training of the SVC model, which depended on manual intervention and a small number of samples. Although the small training set reduced the labeling burden, it also implies that feature recognition is constrained by annotation quality. Across different oilfields, well trajectories, fiber installation methods, coupling conditions, production regimes, and gauge lengths, the morphology of the V-shaped feature may vary. Re-annotation and fine-tuning for new environments may be necessary, increasing the application cost and complexity.

The computational efficiency of the parameter-optimization stage is another limitation. PSO introduced population-level evaluations into an otherwise one-dimensional hyperparameter-selection problem. A deterministic grid search may therefore reduce the optimization cost and improve the reproducibility. The performance of nonlinear kernels was not evaluated, so the effect of kernel choice remains unresolved.

Finally, the entire processing workflow was based on a key premise: that the effective upgoing and downgoing wave signals in the F–K domain form coherent branches separable by the OCSVM decision boundary. In scenarios with an extremely low signal-to-noise ratio (SNR), variable coupling, strong azimuthal sensitivity effects, or dispersed wave modes, the effective signal may be difficult to distinguish from noise, leading to performance degradation [38].

In summary, within the stated assumptions, the proposed model provides a practical solution for low-frequency DAS signal processing in production logging. The study demonstrates how physically interpretable filtering and an F–K-domain wavefield analysis can be combined with data-driven feature extraction for DAS production-logging interpretation. These limitations indicate the need for more adaptive feature extraction, reduced reliance on empirical rules, and validation using independent multi-well datasets. Future evaluation should focus on independent multi-well validation, controlled comparisons with alternative feature-extraction and filtering methods, and an assessment of the sensitivity to the coupling and acquisition parameters.

## 5. Conclusions

This paper proposes a hybrid low-frequency DAS processing framework for distributed optical fiber production logging. A simple FIR preprocessing step suppresses high-frequency fluctuations before downsampling, while the main interpretation workflow integrates 2D FFT, PSO-optimized OCSVM, rule-based enhancement, SVC refinement, and linear regression in the F–K domain. The model comprises preprocessing, feature extraction, feature enhancement, and velocity-fitting modules.

The rule-based approach can remove axis-parallel patterned noise in the F–K domain image, but remains ineffective for noise that deviates from predefined rules. After initial rule-based denoising, the amount of noise is reduced, leaving a limited number of residual points. The SVC model can be trained with a small number of samples to identify these irregular residual points, thereby enhancing feature continuity before linear regression.

Six overlapping validation windows using field DAS data from the investigated field well demonstrated relative errors ranging from 0.08% to 3.13% against field-reference values. These results indicate that the proposed workflow can support distributed flow-velocity estimation for this case study, while further validation is required before extending the approach to other wells, production regimes, or acquisition configurations.

## Figures and Tables

**Table 1:** DAS acquisition configuration used in the field case.

Parameter	Value
Interrogator	Silixa DAS interrogator
Original temporal sampling frequency	10,000 Hz
Temporal downsampling factor	2
Processing sampling frequency	5000 Hz
Temporal segment length	15 s
Spatial sampling rate	2 samples/m
Gauge length	10 m
Fiber sensing length	4400 m
Target interval	4000–4200 m
Production condition	Oil–water two-phase production well
Acquisition timing	Approximately two hours after well opening
Data domain	Time–space DAS response matrix
Data format	Field DAS records in Hierarchical Data Format version 5 (HDF5; .hdf5)

**Table 2:** Parameter settings and optimization results of the particle swarm optimization algorithm.

Parameter Name	Setting Value
Search range for ν	(0, 1]
Population size	100
Iterations	20
Inertia weight	0.7
Individual learning factor	2
Social learning factor	2
Optimal parameter ν	0.954681
Test-set classification accuracy	96.88%
Average computation time	15.12 s/iteration

**Table 3:** Processing stages and corresponding visual evidence in the F–K domain workflow.

Processing Stage	Corresponding Figure	Description
Original F–K domain spectrum	Figure 7a	F–K domain spectrum obtained by applying 2D FFT to the selected time–space matrix.
Centered F–K domain spectrum	Figure 7b	FFT-shifted spectrum with the zero-frequency and zero-wavenumber components moved to the center.
Truncated 0–150 Hz feature view	Figure 8	Low-frequency F–K domain view in which the V-shaped feature becomes more apparent.
OCSVM extraction	Figure 10b	Initial binary V-shaped feature obtained after OCSVM decision-boundary thresholding.
Rule enhancement	Figure 10c	Removal of horizontal and zero-wavenumber axis-parallel streak noise using empirical rules.
SVC refinement	Figure 10d	Removal of sparse residual noise around the V-shaped branches using the trained SVC.

**Table 4:** SVC classification metrics calculated from the confusion matrices in Figure 12.

Segment/Class	Accuracy (%)	Precision (%)	Recall (%)	F1-Score (%)
Left/noisy data	98.59	96.32	91.61	93.91
Left/normal data	98.59	98.88	99.53	99.21
Right/noisy data	97.76	100.00	89.24	94.31
Right/normal data	97.76	97.25	100.00	98.61

**Table 5:** Apparent propagation velocities and 95% confidence intervals obtained from the fitted F–K branches.

Branch	Apparent Velocity (m/s)	95% CI (m/s)
Negative wavenumber	−1559.41	[−1582.38,−1526.44]
Positive wavenumber	1591.33	[1588.76,1593.90]

**Table 6:** Comparison between DAS-calculated and field-reference flow velocities.

Interval (m)	Calculated Velocity (m/s)	Reference Velocity (m/s)	Error (%)
4000–4200	15.96	16	0.25
3900–4100	15.50	16	3.13
3800–4000	14.86	15	0.93
3700–3900	14.36	14	2.57
3600–3800	13.30	13	2.31
3500–3700	13.01	13	0.08

**Table 7:** Sensitivity of velocity estimation to FIR filter length.

Filter Length	Estimated Velocity (m/s)	Relative Error (%)
3	15.96	0.25
5	15.91	0.56
7	15.53	2.94

## Data Availability

The original contributions presented in this study are included in the article. The field DAS data are subject to operational confidentiality restrictions and are not publicly available. Further inquiries can be directed to the corresponding author.

## Abbreviations

The following abbreviations are used in this manuscript:

DAS	Distributed Optical Fiber Acoustic Sensing
FIR	Finite Impulse Response
F–K	Frequency–Wavenumber
FFT	Fast Fourier Transform
DFT	Discrete Fourier Transform
HDF5	Hierarchical Data Format version 5
OCSVM	One-Class Support Vector Machine
PSO	Particle Swarm Optimization
SVC	Support Vector Classifier
LR	Linear Regression

## References

[1] He Z., Liu Q. (2021). Optical Fiber Distributed Acoustic Sensors: A Review. J. Light. Technol..

[2] Sun Q., Fan C., Li H., Yan B., Yan Z., Yu G., Liu D. (2022). Progress of Research on Optical Fiber Distributed Acoustic Sensing Technology in Petroleum Industry. Geophys. Prospect. Pet..

[3] Zhou X., Chen W., Yang J., Li X. (2021). Application Review of DAS Technology in Oil and Gas Geophysics. Sinopec Geophys. Res. Inst..

[4] Ekechukwu G.K., Sharma J. (2021). Well-Scale Demonstration of Distributed Pressure Sensing Using Fiber-Optic DAS and DTS. Sci. Rep..

[5] Shang Y., Sun M., Wang C., Yang J., Du Y., Yi J., Zhao W., Wang Y., Zhao Y., Ni J. (2022). Research Progress in Distributed Acoustic Sensing Techniques. Sensors.

[6] Titov A., Jin G., Binder G., Tura A. (2022). Distributed Acoustic Sensing Time-Lapse Vertical Seismic Profiling during Zipper-Fracturing Operations: Observations, Modeling, and Interpretation. Geophysics.

[7] Booth A.D., Christoffersen P., Schoonman C., Clarke A., Hubbard B., Law R., Doyle S.H., Chudley T.R., Chalari A. (2020). Distributed Acoustic Sensing of Seismic Properties in a Borehole Drilled on a Fast-Flowing Greenlandic Outlet Glacier. Geophys. Res. Lett..

[8] Jousset P., Currenti G., Schwarz B., Chalari A., Tilmann F., Reinsch T., Zuccarello L., Privitera E., Krawczyk C.M. (2022). Fiber Optic Distributed Acoustic Sensing of Volcanic Events. Nat. Commun..

[9] Binder G., Titov A., Liu Y., Simmons J., Tura A., Byerley G., Monk D. (2020). Modeling the Seismic Response of Individual Hydraulic Fracturing Stages Observed in a Time-Lapse Distributed Acoustic Sensing Vertical Seismic Profiling Survey. Geophysics.

[10] Ekechukwu G.K., Sharma J., William M.J. (2023). A Novel Velocity Band Energy Workflow for Fiber-Optic DAS Interpretation and Multiphase Flow Characterization. Sci. Rep..

[11] Sharma J., Santos O.L.A., Feo G., Ogunsanwo O., Williams W. (2020). Well-Scale Multiphase Flow Characterization and Validation Using Distributed Fiber-Optic Sensors for Gas Kick Monitoring. Opt. Express.

[12] Sharma J., Cuny T., Ogunsanwo O., Santos O. (2020). Low-Frequency Distributed Acoustic Sensing for Early Gas Detection in a Wellbore. IEEE Sens. J..

[13] Bublin M. (2021). Event Detection for Distributed Acoustic Sensing: Combining Knowledge-Based, Classical Machine Learning, and Deep Learning Approaches. Sensors.

[14] Wang X.y., Zhang L.m., Zhang K., Cheng C. (2025). Knowledge-Data Synergy Enabling Zero-Shot Composite Fault Diagnosis in Sucker-Rod Pumping Systems. Eng. Appl. Artif. Intell..

[15] Zhang J., Wu M., Hao C., Wu Y. (2025). Wideband Joint Elevation–Azimuth Angle Estimation Based on Multiple Frequency Model and Atomic Norm Minimization. IEEE Trans. Instrum. Meas..

[16] Vahabi N., Willman E., Baghsiahi H., Selviah D.R. (2020). Fluid Flow Velocity Measurement in Active Wells Using Fiber Optic Distributed Acoustic Sensors. IEEE Sens. J..

[17] Williams E.F., Zhan Z., Martins H.F., Fernández-Ruiz M.R., Martín-López S., González-Herráez M., Callies J. (2022). Surface Gravity Wave Interferometry and Ocean Current Monitoring with Ocean-Bottom DAS. J. Geophys. Res. Ocean..

[18] Tariq Z., Aljawad M.S., Hasan A., Murtaza M., Mohammed E., El-Husseiny A., Alarifi S.A., Mahmoud M., Abdulraheem A. (2021). A Systematic Review of Data Science and Machine Learning Applications to the Oil and Gas Industry. J. Pet. Explor. Prod. Technol..

[19] Yang J., Shragge J., Jin G. (2022). Filtering Strategies for Deformation-Rate Distributed Acoustic Sensing. Sensors.

[20] Temizel C., Canbaz C.H., Palabiyik Y., Aydin H., Tran M., Ozyurtkan M.H., Yurukcu M., Johnson P. (2021). A Thorough Review of Machine Learning Applications in Oil and Gas Industry. Proceedings of the SPE/IATMI Asia Pacific Oil and Gas Conference and Exhibition.

[21] Kuang L., Liu H., Ren Y., Luo K., Shi M., Su J., Li X. (2021). Application and Development Trend of Artificial Intelligence in Petroleum Exploration and Development. Pet. Explor. Dev..

[22] Li Y., Li F., Li J., Jin Q., Liu C. (2020). Application of Distributed Acoustic Sensing in Borehole Seismic Exploration. Geophys. Prospect. Pet..

[23] Ting L., Jinfa Z., Yingzhu G., Guojun Y., Qi Z., Qinghui Z. (2023). Progress on Each Section Productivity Evaluation Technology of Horizontal Well Staged Fracturing. Sci. Technol. Eng..

[24] Maulud D., Abdulazeez A.M. (2020). A Review on Linear Regression Comprehensive in Machine Learning. J. Appl. Sci. Technol. Trends.

[25] Zhang D., Liang Y., Sun Z., Mukherjee M. (2021). One-Class Support Vector Machine with Particle Swarm Optimization for Geo-Acoustic Anomaly Detection. Proceedings of the 2021 17th International Conference on Mobility, Sensing and Networking (MSN), Exeter, UK, 13–15 December 2021.

[26] Harper R., Taylor A. (2023). Genetic Algorithm Based Optimization of One-Class Support Vector Machine for Abnormality Detection in Oilfield Centrifugal Pump Injection Stations. Sci. Technol. Eng..

[27] Cuong-Le T., Nghia-Nguyen T., Khatir S., Trong-Nguyen P., Mirjalili S., Nguyen K.D. (2022). An Efficient Approach for Damage Identification Based on Improved Machine Learning Using PSO-SVM. Eng. Comput..

[28] Chen H., Fan D.L., Fang L., Huang W., Huang J., Cao C., Yang L., He Y., Zeng L. (2020). Particle Swarm Optimization Algorithm with Mutation Operator for Particle Filter Noise Reduction in Mechanical Fault Diagnosis. Int. J. Pattern Recognit. Artif. Intell..

[29] Araujo J.G., Brito H.G., Galvao M.V., Maitelli C.W.S.P., Doria Neto A.D. (2025). Deep Learning Models Applied for Flowrate Estimation in Offshore Wells with Electric Submersible Pump. Energies.

[30] Ashry I., Mao Y., Wang B., Hveding F., Bukhamsin A., Ng T.K., Ooi B.S. (2022). A Review of Distributed Fiber-Optic Sensing in the Oil and Gas Industry. J. Light. Technol..

[31] Raiaan M.A.K., Sakib S., Fahad N.M., Mamun A.A., Rahman M.A., Shatabda S., Mukta M.S.H. (2024). A Systematic Review of Hyperparameter Optimization Techniques in Convolutional Neural Networks. Decis. Anal. J..

[32] Kushnir U., Frid V. (2023). Spectrum-Based Logistic Regression Modeling for the Sea Bottom Soil Categorization. Appl. Sci..

[33] Kwon D., Kim H., Kim J., Suh S.C., Kim I., Kim K.J. (2019). A Survey of Deep Learning-Based Network Anomaly Detection. Clust. Comput..

[34] Hu J., Wang Y. (2025). Wellbore Fluid Flow Velocity Calculation Based on Distributed Acoustic Sensing Data. J. Geophys. Eng..

[35] Lu P., Lalam N., Badar M., Liu B., Chorpening B.T., Buric M.P., Ohodnicki P.R. (2019). Distributed Optical Fiber Sensing: Review and Perspective. Appl. Phys. Rev..

[36] Shipe M.E., Deppen S.A., Farjah F., Grogan E.L. (2019). Developing Prediction Models for Clinical Use Using Logistic Regression: An Overview. J. Thorac. Dis..

[37] Wu Y., Guo H. (2024). Oil-Water Flowrate Measurement with Sensing Data and Equidistant Area-Weighted Average Method. Flow Meas. Instrum..

[38] Zhang X., Xie X., Tang S., Zhao H., Shi X., Wang L., Wu H., Xiang P. (2024). High-Speed Railway Seismic Response Prediction Using CNN-LSTM Hybrid Neural Network. J. Civ. Struct. Health Monit..

